# New Medical Journals

**Published:** 1879-03

**Authors:** 


### ARTICLE XVI.

### NEW MEDICAL JOURNALS.

New medical periodicals have been appearing so frequently that it had become a matter of little comment with us to find a new specimen copy upon our table every few months. Many of them lived but a short time; their ephemeral existence ceased before the ink which announced their appearance to the reader had fairly dried.

A study of the mortality statistics of American medical periodicals can easily detect the principal cause of the high death-rate among them. The *causa proxima*, of course, is want of patronage. The subscription list remains so small that the publisher is soon convinced, continuation of his medical journal is equivalent to sacrificing so many hundreds or thousands of dollars upon the altar of medical journalism. And as his charity does not reach this height of enthusiasm, the publication is suspended.

But from the scanty patronage of many periodicals it must by no means be inferred that American physicians lack the profoundest interest in the progress of that science to which they have devoted their life. This conclusion would be entirely fallacious. On the contrary, we believe reading of periodical medical literature is more universal with the profession of this country than with that of any other nation; we know to an American physician a medical journal is as necessary a companion as the daily newspaper to every American citizen.

The trouble is, too many medical papers are published, not to supply a real want, but for purposes altogether foreign to, and incompatible with the dignity of scientific journalism. But the body medical is quick in discriminating between cheap, worthless publications and meritorious medical journals. It supports the latter and rejects the others.

New medical periodicals of lasting value will be brought out only when and where the increasing number of scientific workers develops a natural demand for new channels through which they can communicate to the profession the results of their investigations and observations. Such periodicals are always welcome and will not fail to receive the hearty support of the medical profession.

We have recently received a few new exchanges of this kind, which we wish to recommend very warmly to the attention of our readers. Their *raison d'être* is the growing scientific activity in the American medical profession; their *aim* is solely the advancement of medical science; their *standard* is of the highest order and equal to the best medical periodicals of Europe.

We will give our readers a brief description of these new journals:

1. INDEX MEDICUS, A MONTHLY CLASSIFIED RECORD OF THE CURRENT MEDICAL LITERATURE OF THE WORLD. This is the title of a new periodical, edited by Dr. John S. Billings, Surgeon U. S. Army, and Dr. Robert Fletcher, M. R. C. S., England. The January number is before us, and it surpasses the most sanguine expectations which could be excited by the pretentious promise held out in the prospectus that it should record the medical literature of the world. The list of medical periodicals alone which are received by the editors fills twenty-four quarto pages. The remaining forty-eight pages of the number contain a list of all books, pamphlets and original articles published during the last quarter of the past year. The subjects of this index are arranged under the following heads: Anatomy and Physiology, with five subdivisions; Medicine, with sixteen subdivisions; Therapeutics and Materia Medica, with four subdivisions; Surgery, with ten subdivisions; Gynæcology; Obstetrics; Diseases of Children; Ophthalmology; Otology and Diseases of the Nose and Throat; Diseases of the Teeth; State Medicine; Veterinary Medicine, Miscellaneous. It is scarcely necessary to demonstrate the immense value of such a work for any practitioner, teacher and writer. In its pages he will find the titles of parallels for his own observations, accounts of new remedies, the latest methods of therapeutics and full information of what has been written by others on any subject he may wish to investigate.

It is a gigantic task, conceived and executed by that same energy of American enterprise which has built the Pacific Railroad, tunneled our lakes and realized in the telephone the most fanciful of dreams.

The INDEX is published by T. Leypoldt, 37 Park Row, New York; subscription three dollars per annum.

2 Another very important addition to American periodical medical literature is the AMERICAN JOURNAL OF OTOLOGY, edited by Clarence J. Blake in conjunction with other aural surgeons of national reputation. It is a quarterly journal of about eighty pages, intended as a medium for the publication of original communications on physiological acoustics and aural surgery, also to furnish carefully prepared reviews of books treating of subjects pertaining to acoustics and diseases of the ear, and to give a digest of the more important papers on aural surgery, published in other periodicals and in reports of medical societies.

The fact that the American text-books on diseases of the ear are acknowledged to be the best works of this kind in the English language; the recognition all the world over of the transactions of the American Otological Society as valuable scientific contributions; and the high standing of the editors of this journal, are a sufficient guaranty for its success, as a promoter and exponent of science. Whether it will be a success for the publishers depends solely upon the interest the American medical profession will show for it by a liberal patronage, which it justly deserves. It is published by William Wood & Co., 27 Great Jones street, N. Y. Price, three dollars per annum.

3. The ARCHIVES OF MEDICINE, edited by E. C. Seguin, M.D., in connection with other gentlemen of high standing, may well be mentioned side by side with its other two new American contemporaries. Its pages are open for scientific original communications and observations upon matters relating to medicine; some space also is allowed for book reviews and summary. The first number is a fine specimen of medical Journalism, both as to the reading matter and to the press work.

The truly scientific spirit which pervades the pages of the *Archives* reminds us strongly of the *Archiv. der Heilkunde*, which, after 20 years of existence, had to be suspended for want of patronage. Let us hope this journal will meet with a better fate than its German namesake. It is to be a bi-monthly journal, published by G. P. Putnam's Sons, 182 Fifth avenue, N. Y. Subscription price, three dollars per year.

4. With the foregoing periodicals we wish to rank a publication which was started last year by the united efforts of a number of English and American scientists, and which deserves the support of all physicians whose interest is not limited to reading on strictly practical subjects. Physicians, then, who in their spare moments like to learn what is going on in the collateral sciences, will find enjoyment in reading the JOURNAL OF PHYSIOLOGY, edited by Michael Foster, M.D., in conjunction with others, and published in London and New York, 22 Bond Street, by Macmillan & Co. From four to six numbers will form a volume of about 500 pages. Subscription price, $5.25.

---

A CONFESSIONAL.—The *British Medical Journal* has established in its columns a confessional department, in which practitioners are invited to report failures, blunders and errors of all kinds.

The idea is such an excellent one that we hasten to profit by it. We invite all our readers who have to confess mistake, carelessness, ignorance or unavoidable failure, to contribute to such a department in this journal—and that whether they be repentant or unrepentant sinners. Such as may desire to append their names to their communications may do so, not for publication, but as a guarantee of good faith, the editors holding themselves strictly responsible for inviolable secrecy upon that point. Communications without names will be received and published if they bear internal evidence of truthfulness.

We are confident that a mistake, failure or blunder may thus be made to accomplish in the end a great deal of good.

